# Integrating clinicopathologic factors with whole-exome sequencing and peripheral immune repertoire sequencing to optimize decision-making in neoadjuvant chemoimmunotherapy for HNSCC

**DOI:** 10.3389/fimmu.2026.1821734

**Published:** 2026-07-30

**Authors:** Yi-Wei Zhong, Pu-Gen An, Xiao Hu, Lei Zheng, Dong-Sheng Yu, Jie Zhang, Wen-Jie Wu

**Affiliations:** 1 Hospital of Stomatology, Guanghua School of Stomatology, Sun Yat-sen University, Guangzhou, China; 2 Guangdong Provincial Key Laboratory of Stomatology, Sun Yat-sen University, Guangzhou, China; 3 Department of Oral and Maxillofacial Surgery, Peking University School and Hospital of Stomatology, Beijing, China; 4 National Center of Stomatology & National Clinical Research Center for Oral Diseases, Beijing, China; 5 Central Laboratory, Peking University School and Hospital of Stomatology, Beijing, China; 6 Peking University Hospital of Stomatology Sanya Division (Sanya Stomatology Center), Sanya, China

**Keywords:** biomarker, de-escalation surgery, head and neck squamous cell carcinoma, immune repertoire sequencing, neoadjuvant chemoimmunotherapy

## Abstract

**Background:**

Neoadjuvant chemoimmunotherapy (NACIT) improves survival in patients with locally advanced head and neck squamous cell carcinoma (HNSCC), but optimal decisions remain challenging. Clinicopathological factors combined with whole-exome sequencing (WES) and peripheral immune repertoire sequencing (IRS) are promising for identifying prognostic factors to provide insights for clinical decisions and underlying mechanisms.

**Methods:**

Patients with locally advanced HNSCC treated with NACIT across 3 studies were included. Clinicopathological factors labeled with different time points, integrated with WES mutation features, peripheral IRS indices and clonotypes, were analyzed. The endpoints were overall survival (OS), event-free survival (EFS), and major pathological response (MPR). Multivariate logistic regression and Cox proportional hazards models were used to identify independent prognostic factors. Correlation analyses were used to explore the associations between sequencing indices and clinicopathological characteristics.

**Results:**

A total of 101 patients were included, with a median follow-up of 38 months, (from 2 to 55 months). Pathology revealed 50 (49.5%) patients with an MPR and 32 (31.7%) with a pathological complete response (pCR). The 3-year OS and EFS rates were 69.4% and 60.1%, respectively. Clinically, performance status (PS) was found to be associated with not only survival but also response. A positive surgical margin caused by extranodal extension significantly affects survival. Biologically, the IRS model based on specific T-cell and B-cell clonotypes demonstrated potential utility in predicting prognosis. High immune repertoire diversity and clonality were significantly correlated with better PS and primary tumor. The WES model showed limited prognostic utility while patients with *PABPC1* mutation had higher MPR rate.

**Conclusion:**

Clinically, the PS was related to response and survival. ENE associated positive surgical margins remain a threat to survival. Biologically, the IRS model shows preliminary potential for patient stratification, warranting further investigation. Correlations of increased immune diversity with better physical status and primary tumor support earlier NACIT and are worthy of further exploration.

## Introduction

Strategies for managing head and neck squamous cell carcinoma (HNSCC) have gradually advanced with the development of immune checkpoint inhibitors (ICIs) (1). Historically, despite the use of aggressive therapies, patients with HNSCC still suffer from poor survival and significant morbidity (2, 3). Recently, a series of trials and studies on neoadjuvant chemoimmunotherapy (NACIT) for HNSCC have demonstrated its advantages over upfront surgery (2, 4, 5). However, given the distinct anatomical features of the head and neck region, controversies persist regarding key determinants of clinical decision-making, including the initiation of NACIT, surgical strategies, and the choice of adjuvant therapy (4, 6, 7).

Numerous clinicopathological factors and biomarkers related to response or survival have been reported (8–11). Among these, programmed death-ligand 1 (PD-L1) expression (8), radiological response (12), and pathological response (13) are the most widely accepted biomarkers in clinical practice. However, only PD-L1 expression was used to determine whether a patient should undergo NACIT, and there were still a certain portion of HNSCC patients missed accurate assessment (14). And also, radiological evaluation of surgical extent lacks reliability since tumor regression patterns vary (15). Furthermore, even pathological complete response (pCR) could not spare the patient from requiring postoperative radiotherapy. In most of the above studies for biomarkers, fewer endpoint events occurred because of the strict inclusion criteria, and factors from different time points were combined in multivariate analyses. Owing to these factors, less significant results may have been obtained. Thus, to improve clinical decision-making, more stable and accurate clinicopathological factors to identify appropriate candidates are needed.

Activation of specific T-cell and B-cell clones is the core mechanism of immunotherapy, and immune repertoire sequencing (IRS) can be used to capture clonal expansion and replacement data (16, 17). Compared with whole-exome sequencing (WES) focusing on mutation burden and PD-L1 expression focusing on the target, IRS can directly reflect the activation of the adaptive immune system and its capacity for tumor recognition (16, 18). Furthermore, the T-cell repertoire in peripheral blood has been regarded as a potential biomarker for anti-programmed cell death protein-1 (PD-1) therapy in patients with HNSCC (19, 20). Recent evidence has suggested that B-cell receptor (BCR) diversity and cooperation among T and B cells are both also critical for achieving anti-PD-1 responses (17). Accordingly, integrating both T-cell receptor (TCR) and BCR clonotype dynamics, as well as WES patterns, with clinicopathological factors might represent a promising strategy to imply the mechanism underlying NACIT in patients with HNSCC.

Therefore, in this study, we proposed a pooled analysis approach and integrated time point-labeled clinicopathological factors with peripheral IRS and WES data to evaluate their relationships with response and survival. The aim was to provide preliminary insights to support clinical decisions in different situations, present first hints regarding the immune mechanism of NACIT, and explore the preliminary utility of peripheral IRS and WES for patient stratification.

## Materials and methods

### Study population

This study was conducted on the basis of 1 retrospective cohort (cohort 1) (6) and 2 registered prospective clinical trials (cohort 2 with ChiCTR2200056354 and cohort 3 with ChiCTR2100054296) (21, 22), and patients from these studies with WES and/or IRS data were included. All of the treatments for locally advanced oral or oropharyngeal cancer treated with NACIT using tislelizumab, albumin-bound paclitaxel, and cisplatin were designed similarly. Detailed treatment protocols are presented in previous studies or on a registry website (Chictr.org.cn).

### Related factor data collection

Factors potentially related to response or survival were derived from the original medical history and included sex (defined as the biological sex), age, Eastern Cooperative Oncology Group performance status (PS), smoking status, alcohol status, site of the primary tumor (classified as oral or oropharyngeal), HPV status of oropharyngeal cases, sum of the diameters of the baseline target lesions, tumor proportion score (TPS), combined positive score (CPS), extranodal extension, c(r)TNM, sum of the diameters of the preoperative target lesions, radiological response, surgical strategy, yc(r)TNM, surgical margin, pathological response, and postoperative therapy.

Responses were assessed on the basis of the Response Evaluation Criteria in Solid Tumors (RECIST1.1). Major pathological response (MPR) was defined as ≤10% residual tumor cells in the primary tumor and lymph nodes involved. pCR was defined as the absence of residual tumor cells in the primary tumor and lymph nodes involved. PD-L1 expression was measured via a PD-L1 IHC 22C3 PharmDx Assay Kit (Agilent Technologies, Santa Clara, CA, USA) according to the manufacturer’s instructions. The CPS was defined as the number of PD-L1-positive cells (tumor cells, lymphocytes, and macrophages) divided by the total number of tumor cells × 100. TPS was defined as the number of PD-L1-positive tumor cells divided by the total number of tumor cells × 100%. TNM stage was assessed according to the American Joint Committee on Cancer Staging Manual, eighth edition. All the factors above were classified as baseline, preoperative, and postoperative according to their collection times. These time points were defined as followed: baseline was the period within one week prior to the initiation of NACIT; preoperative was the period after completion of NACIT but before surgery; postoperative was the period following surgery.

The endpoints of this study were overall survival (OS, defined as the period from neoadjuvant therapy to the latest follow-up or death from any cause during follow-up) and event-free survival (EFS, defined as the period from neoadjuvant therapy to the latest follow-up, recurrence, distant metastasis, or death from any cause during follow-up).

### Whole-exome sequencing

Tumor samples and paired peripheral blood samples from the patients were collected at baseline, and genomic DNA was extracted using a library extraction kit (MyGenostics, Inc.) according to the instructions. First, a minimum of 3 µg of DNA was used for the indexed Illumina libraries according to the manufacturer’s protocol (MyGenostics, Beijing). DNA fragments with sizes ranging from 350 bp to 450 bp and those including the adapter sequences were selected for the DNA libraries. The final product was validated using a Nanodrop 2000 (Thermo Fisher, USA) and an Agilent Bioanalyzer (Agilent, USA). The whole coding exons of genes were subsequently selected via a gene capture strategy using the GenCap custom enrichment kit (MyGenostics, Beijing) following the manufacturer’s protocol. The enriched libraries were subsequently sequenced on an Illumina NextSeq 500 sequencer (Illumina, San Diego, CA, USA) for 150 bp paired-end reads. Finally, following sequencing, low-quality variations were filtered out using a quality score ≥20, and Burrows–Wheeler Aligner software was used to align the clean reads to the reference human genome (hg19). Single-nucleotide polymorphisms, insertions and deletions were identified using Genome Analysis Toolkit software. Structural variations were determined using Delly. Copy number variants were detected using CNVkit, and copy number changes were detected on the basis of the depth distribution of the reads compared with the reference genome.

### Peripheral immune repertoire sequencing

Peripheral blood samples were obtained from the patients within one week prior to the initiation of NACIT, and genomic DNA was extracted. The complementarity-determining region 3 (CDR3) sequences of the TCRβ chain and BCR heavy chain were amplified through multiplex PCR and sequenced on the Illumina HiSeq2500 platform (MyGenostics, Beijing, China). Here, 1 μg of genomic DNA from each sample was used. The CDR3 sequences of the TCRβ chain or BCR heavy chain were aligned on the basis of the definition provided by the International ImMunoGeneTics collaboration (23). Unmatched sequencing reads were subsequently filtered out. A standard algorithm was applied to identify the V, D, and J segments contributing to the CDR3 region (23). Since sequences with frameshifts or stop codons cannot be translated into functional proteins, only productive reads that did not include frameshifts or stop codons were included for subsequent downstream analyses.

To comprehensively evaluate the heterogeneity of the TCR/BCR repertoire, we employed distinct metrics quantifying both diversity and clonality. The d50 index, which served as a measure of repertoire evenness, was calculated as the number of unique clonotypes accounting for 50% of the total sequences. Global diversity was further assessed using the inverse Simpson index. To characterize clonal expansion, we calculated the clonal proportion, defined as the cumulative frequency of the top 1% and top 10% most abundant clonotypes.

### Data processing and statistical analysis

The entire process was conducted using R programming (v4.2.1). The median follow-up period for the entire study cohort was calculated according to the reverse Kaplan–Meier method. The chi-square test and Wilcoxon test were used to compare factors between patients in the primary and recurrent groups.

For the WES data, we utilized the data representing the presence or absence of mutations in each gene. The chi-square test was applied, and genes with a *p* value less than 0.05 were selected. The least absolute shrinkage and selection operator algorithm with 10-fold cross-validation was used to select features from the mutated genes in the WES data. Additionally, a logistic regression prediction model for pathological response was constructed on the basis of the mutated genes in the WES data, and the model was validated using the leave-one-out test method. The median risk score was defined as the cutoff value for distinguishing high-risk and low-risk groups and was subsequently analyzed as a related factor. Additionally, the top 20 most frequent mutations were analyzed to evaluate their association with pathological response, OS, or EFS.

With respect to the IRS data, matrix transformation was conducted on the TCR and BCR data. The exact Wilcoxon test with continuity correction was adopted, and VGenes and JGenes with a *p* value less than 0.05 were chosen; among them, those with a fold change between 1.2 and 0.8 were removed. The data were processed like the WES data, as described above. Afterward, the patients with available IRS data were divided into high-risk and low-risk groups, and the risk score was analyzed as a related factor. In addition, the d50 index, inverse Simpson index, top 1% clonal proportion and top 10% clonal proportion were included in correlation analyses with failure to achieve event-free survival, and potentially relevant factors were adjusted for in the multivariate analysis.

Univariate and multivariate logistic regression prediction models and Cox proportional hazards models were used to evaluate the impact of related factors on pathological response, EFS, and OS. The factors included in the multivariate model were chosen on the basis of clinical relevance, previous studies, and relationships in the univariate analysis (p ≤ 0.1). Related factors at different time points were included in different multivariate analyses for various clinical scenarios.

## Results

**Figure 1:**
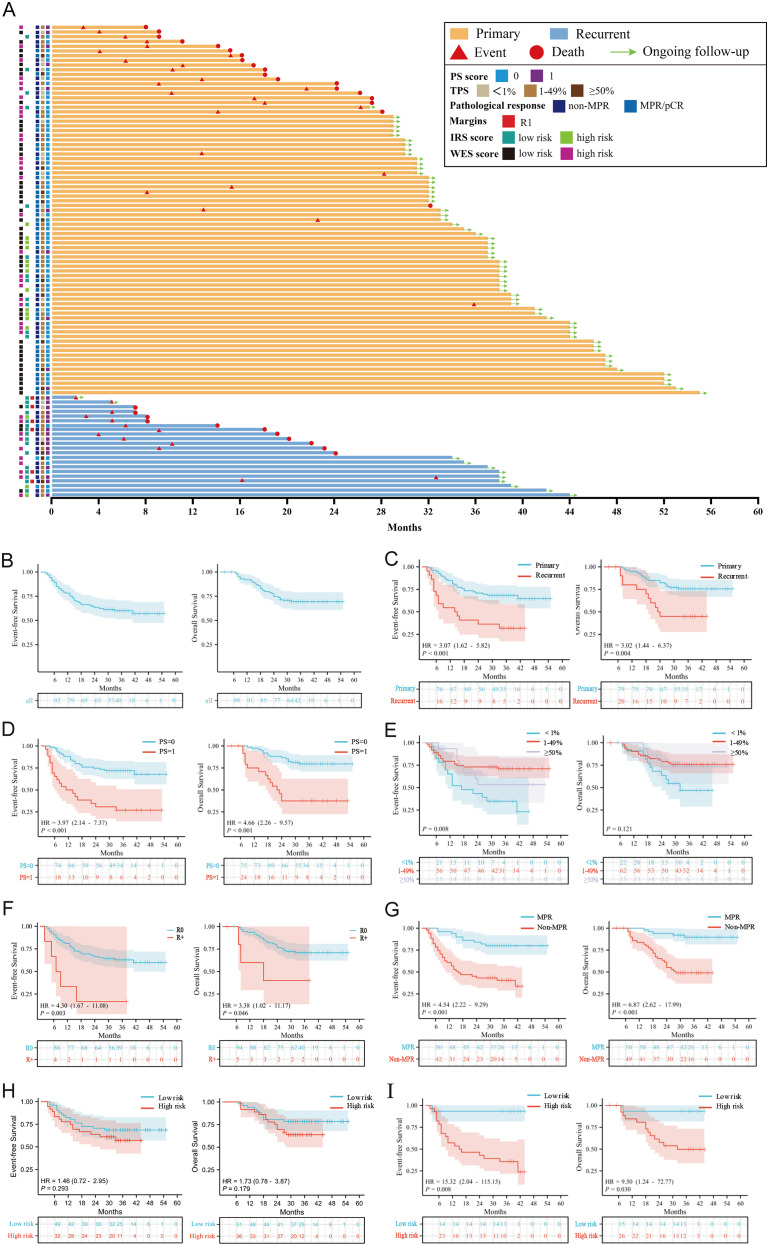
Swimmer plot and Kaplan–Meier survival curves. Swimmer plot of all included patients divided into primary and recurrent groups is shown **(A)**, while each lane represents one patient, and related factors are shown on the left. Kaplan–Meier survival curves showing the EFS and OS of the whole cohort **(B)**, and differences between primary and recurrent tumors **(C)**, between performance statuses of 0 and 1 **(D)**, among tumor proportion scores (TPSs) of <1%, 1~49%, and ≥50% **(E)**, between R0 and R+ initial surgical margins **(F)**, between pathological responses **(G)**, between high- and low-risk patients according to the whole-exome sequencing model **(H)**, and between high- and low-risk patients according to the immune repertoire sequencing model **(I)**.

A total of 101 patients were included in the present study (**Figure 1A**). Among these patients, 87 samples of WES data and 43 samples of IRS data were collected. Until November 2025, the median follow-up period was 38 months (range: 2–55 months). Details of the related factors of the patients at different time points are shown in **Table 1**, and the patients were divided into all, primary, and recurrent groups. Details of the related factors of the patients divided into different cohorts are shown in **Supplementary Table 1**.

**Table 1:** Related factors of the patients at different timepoint.

Time point	Factors	All (n=101)	Primary (n=80)	Recurrent (n=21)	*p*
Baseline	**Sex**				0.198
	Female	19 (18.8%)	13 (16.3%)	6 (28.6%)	
	Male	82 (81.1%)	67 (83.8%)	15 (71.4%)	
	**Age**	58.871 ± 9.523	59.463 ± 9.029	56.619 ± 11.169	0.225
	**Performance status**				**<0.001**
	0	75 (74.2%)	70 (87.5%)	5 (23.8%)	
	1	26 (25.7%)	10 (12.5%)	16 (76.2%)	
	**Smoking**				0.202
	No	29 (32.2%)	24 (30.0%)	5 (50.0%)	
	Yes	61 (67.8%)	56 (70.0%)	5 (50.0%)	
	**Alcohol**				0.236
	No	30 (33.3%)	25 (31.3%)	5 (50.0%)	
	Yes	60 (66.7%)	55 (68.8%)	5 (50.0%)	
	**Sites**				0.135
	Oral	68 (67.3%)	51 (63.7%)	17 (81.0%)	
	Oropharyngeal	33 (32.6%)	29 (36.3%)	4 (19.0%)	
	HPV status*				0.221
	Negative	29 (87.9%)	26 (89.7%)	3 (75.0%)	
	Positive	4 (12.1%)	3 (10.3%)	1 (25.0%)	
	**Sum of the diameters of the baseline target lesions (cm)**	6.643 ± 4.792	7.291 ± 5.120	4.176 ± 1.788	**<0.001**
	**TPS**				0.540
	<1%	23 (22.7%)	20 (25.0%)	3 (14.3%)	
	1~49%	63 (62.3%)	49 (61.3%)	14 (66.7%)	
	≥50%	15 (14.8%)	11 (13.8%)	4 (19.0%)	
	**CPS**				0.072
	<1	6 (5.9%)	5 (6.3%)	1 (4.8%)	
	1~19	63 (62.3%)	54 (67.5%)	9 (42.9%)	
	≥20	32 (31.6%)	21 (26.3%)	11 (52.4%)	
	**ENE**				**0.028**
	No	64 (63.3%)	55 (68.8%)	9 (42.9%)	
	Yes	37 (36.6%)	25 (31.3%)	12 (57.1%)	
	**c (r)TNM**				
	T0	8 (7.9%)	0 (0.0%)	8 (38.1%)	**<0.001**
	T1	5 (5.0%)	4 (5.0%)	1 (4.8%)	
	T2	4 (4.0%)	2 (2.6%)	2 (9.5%)	
	T3	20 (19.8%)	17 (21.3%)	3 (14.3%)	
	T4 or T4a	57 (56.4%)	50 (62.5%)	7 (33.3%)	
	T4b	7 (6.9%)	7 (8.8%)	0 (0.0%)	
	N0	46 (45.5%)	37 (46.3%)	9 (42.9%)	0.050
	N1	9 (8.9%)	9 (11.3%)	0 (0.0%)	
	N2	9 (8.9%)	9 (11.3%)	0 (0.0%)	
	N3	37 (36.6%)	25 (31.3%)	12 (57.1%)	
	III	23 (22.8%)	20 (25.0%)	3 (14.3%)	<0.001
	IV or IVA	51 (50.5%)	33 (41.3%)	18 (85.7%)	
	IVB	27 (26.7%)	27 (33.8%)	0 (0.0%)	
	**WES Score**				**0.005**
	Low risk	51 (58.6%)	48 (64.9%)	3 (23.1%)	
	High risk	36 (41.4%)	26 (35.1%)	10 (76.9%)	
	**IRS Score**				**0.033**
	Low risk	15 (34.9%)	12 (48.0%)	3 (16.7%)	
	High risk	28 (65.1%)	13 (52.0%)	15 (83.3%)	
Preoperative	**Sum of the diameters of the preoperative target lesions (cm)**	3.865 ± 3.231	4.004 ± 3.486	3.200 ± 1.901	0.291
	**Radiological response**				**<0.001**
	Objective response	66 (65.3%)	59 (73.8%)	7 (33.3%)	
	Progressive or stable disease	35 (34.6%)	21 (26.3%)	14 (66.7%)	
	**Surgical strategy**				**<0.001**
	Deescalation surgery	43 (42.6%)	43 (53.8%)	0 (0.0%)	
	Radical surgery	58 (57.4%)	37 (46.3%)	21 (100.0%)	
	**yc (r)TNM**				
	T0	21 (20.7%)	11 (13.8%)	10 (47.6%)	**0.009**
	T1	16 (15.8%)	14 (17.5%)	2 (9.5%)	
	T2	17 (16.8%)	13 (16.3%)	4 (19.0%)	
	T3	16 (15.8%)	16 (20.0%)	0 (0.0%)	
	T4 or T4a	27 (26.7%)	22 (27.5%)	5 (23.8%)	
	T4b	4 (3.9%)	4 (5.0%)	0 (0.0%)	
	N0	53 (52.4%)	46 (57.5%)	7 (33.3%)	0.071
	N1	13 (12.8%)	7 (8.8%)	6 (28.6%)	
	N2	13 (12.8%)	10 (12.5%)	3 (14.3%)	
	N3	22 (21.7%)	17 (21.3%)	5 (23.8%)	
	0	7 (6.9%)	7 (8.8%)	0 (0.0%)	0.667
	I	9 (8.9%)	8 (10.0%)	1 (4.8%)	
	II	10 (9.9%)	8 (10.0%)	2 (9.5%)	
	III	22 (21.7%)	17 (21.3%)	5 (23.8%)	
	IVorIVA	29 (28.7%)	21 (26.3%)	8 (38.1%)	
	IVB	24 (23.7%)	19 (23.8%)	5 (23.8%)	
Postoperative	**Initial surgical margin**				**<0.001**
	R0	95 (94.1%)	80 (100%)	15 (71.4%)	
	R+	6 (5.9%)	0 (0.0%)	6 (28.6%)	
	**Pathological response**				<0.001
	MPR or pCR	50 (49.5%)	48 (60.0%)	2 (9.5%)	
	Neither	51 (50.5%)	32 (40.0%)	19 (90.5%)	
	**Postoperative therapy**				**<0.001**
	Radiotherapy with or without chemotherapy	68 (67.3%)	68 (85.0%)	0 (0.0%)	
	Immunotherapy	17 (16.8%)	3 (3.8%)	14 (66.7%)	
	Watchful waiting	16 (15.8%)	9 (11.3%)	7 (33.3%)	

*In oropharyngeal cases. Bold values indicate statistical significance at p < 0.05.

After receiving neoadjuvant therapy, all the patients successfully underwent surgery. Pathology revealed 50 (49.5%) patients with MPR, among which 32 (31.7%) achieved a pCR. In the primary and recurrent groups, 48 patients (60.0%) and 2 patients (9.5%) had an MPR, respectively, among whom 30 (37.5%) and 2 achieved a pCR, respectively. During the follow-up period, among all the patients, the EFS rates at 1 year, 2 years, and 3 years were 78.2%, 63.4%, and 60.1%, respectively (**Figure 1B**). Among the patients in the primary and recurrent groups, the EFS rates at 1 year, 2 years, and 3 years were 83.8%, 70.0%, and 67.5% and 57.1%, 38.1%, and 33.3%, respectively (**Figure 1C**). Among all the patients, the OS rates at 1 year, 2 years, and 3 years were 91.9%, 74.7%, and 69.4%, respectively (**Figure 1B**). Among the patients in the primary and recurrent groups, the OS rates at 1 year, 2 years, and 3 years were 95.0%, 81.3%, and 74.6% and 78.9%, 47.4%, and 47.4%, respectively (**Figure 1C**).

**Figure 2:**
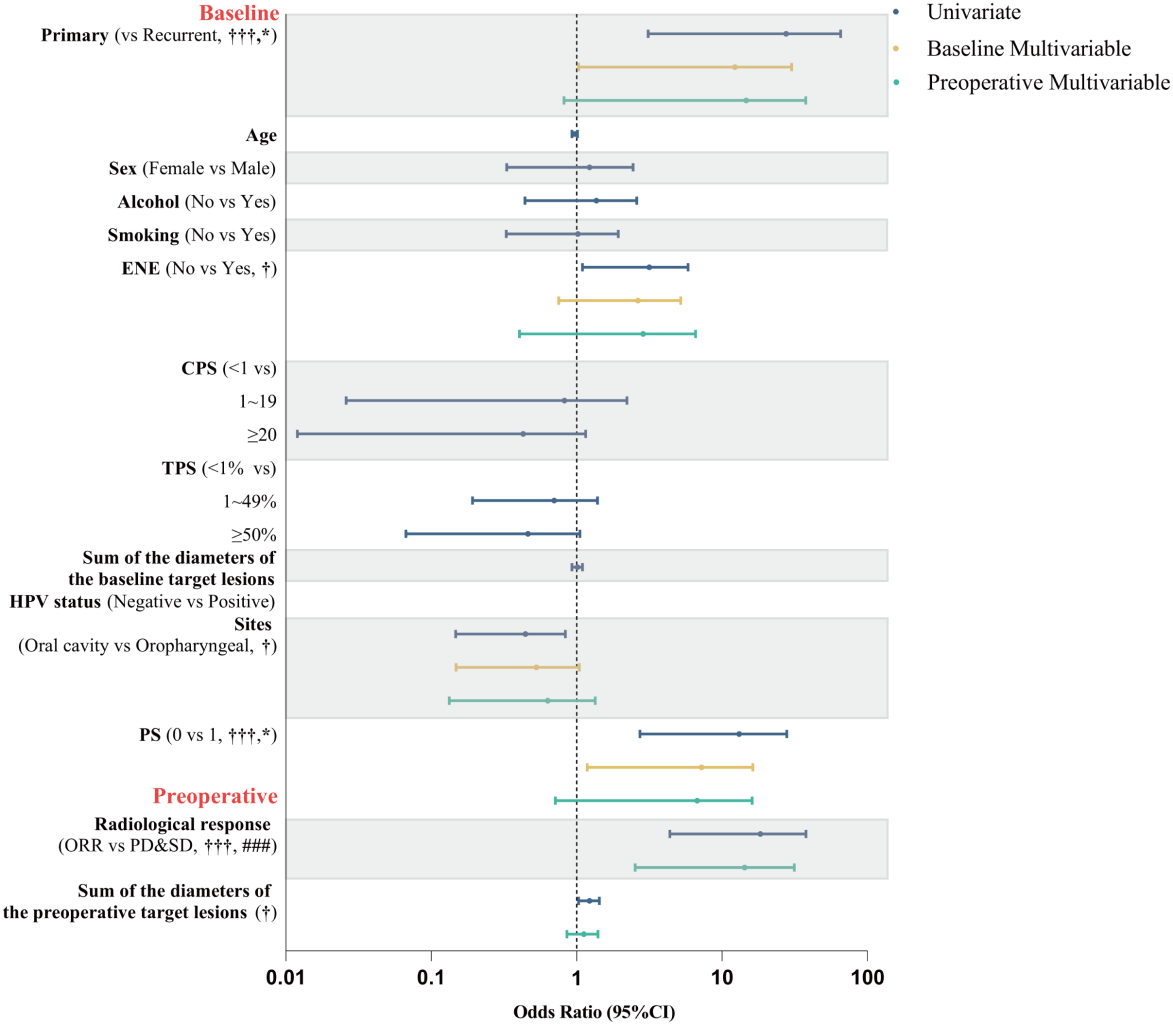
Combined forest plot of factors associated with major pathological response (MPR) in the univariate and multivariate analyses at multiple time points. ^†^(univariate), *(baseline multivariate), and ^#^(preoperative multivariate) indicate statistical significance, where a single symbol represents p<0.05, two symbols represent p<0.01, and three symbols represent p<0.001.

The results of the univariate and multivariate analyses of pathological responses are summarized in **Figure 2**. After multivariate adjustment, at baseline, patients with higher PS scores and recurrent tumor were found to have a greater risk of pathological progressive or stable disease (PD/SD). Patients with radiological PD/SD at the preoperative time point also had a higher risk of pathological PD/SD.

**Figure 3:**
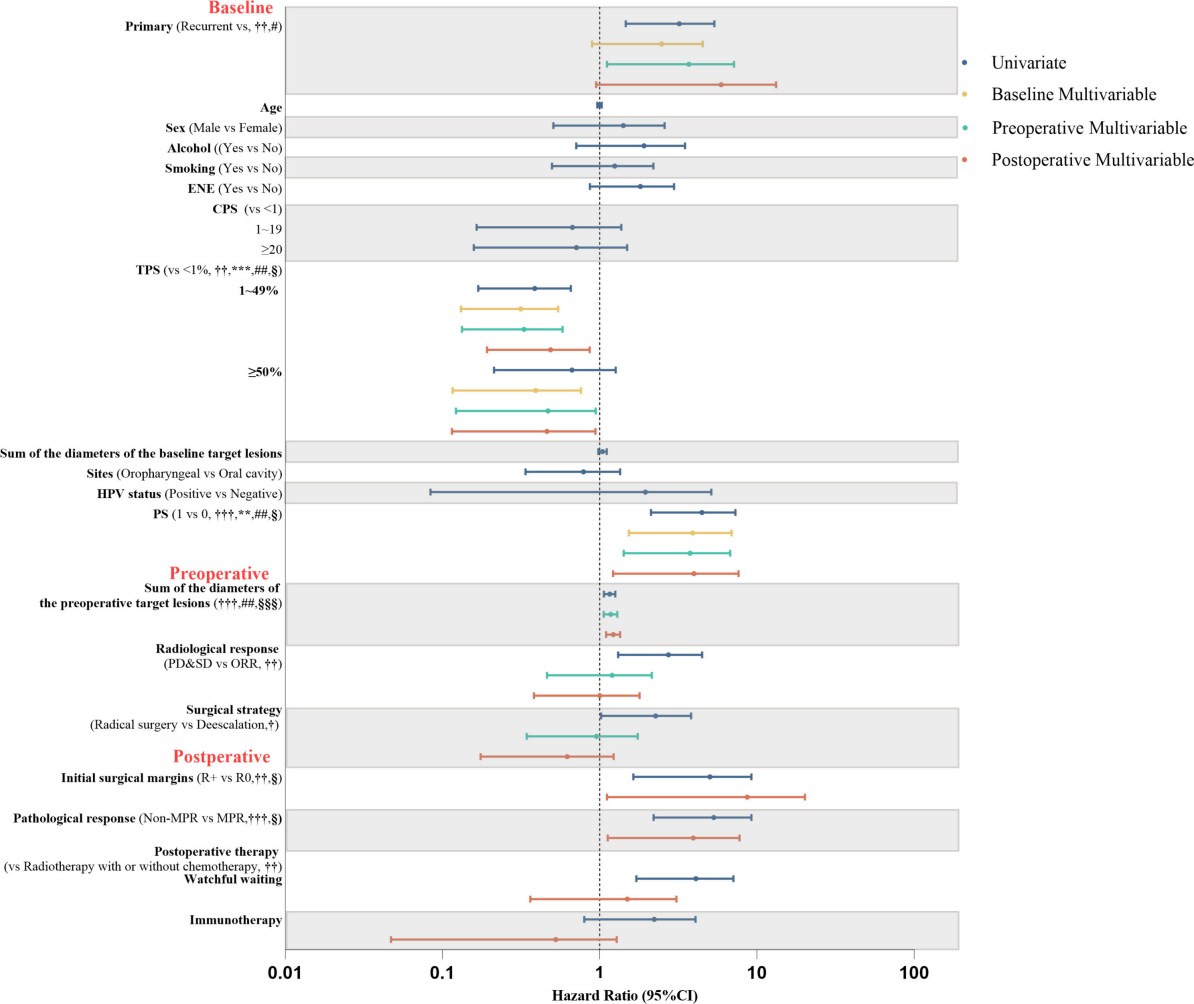
Combined forest plot of factors associated with event-free survival (EFS) in the univariate and multivariate analyses at multiple time points. ^†^(univariate), *(baseline multivariate), ^#^(preoperative multivariate), and ^§^(postoperative multivariate) indicate statistical significance, while a single symbol represents p<0.05, two symbols represent p<0.01, and three symbols represent p<0.001.

The results of the univariate and multivariate analyses of EFS are summarized in **Figure 3**. After multivariate adjustment, patients with high PS scores or low TPSs (**Figures 1D, E**) were found to have a significantly high risk of failure to achieve EFS at all three time points, while recurrent lesions had a similar risk at the preoperative time point. In addition, the sum of the diameters of preoperative target lesions was found to be a significant factor affecting EFS at both the preoperative and postoperative time points. Positive surgical margins (R+) and pathological response were also found to be significant factors affecting EFS at the postoperative time point (**Figures 1F, G**). Importantly, all 6 R+ cases were in the recurrent group, in which carotid artery involvement due to ENE was identified. In these cases, margins remained positive in both frozen and final paraffin sections despite attempts at maximal safe resection.

**Figure 4:**
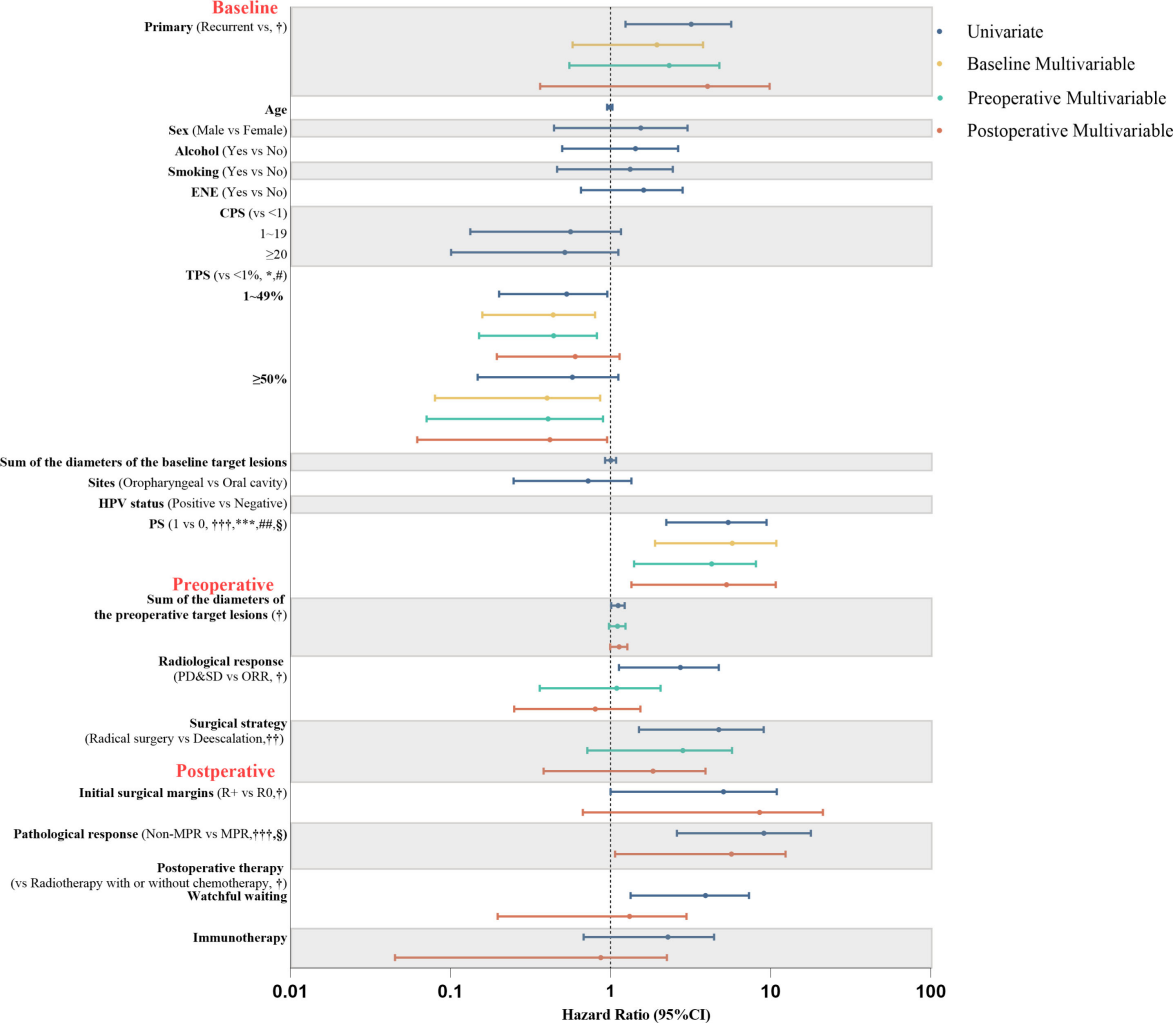
Combined forest plot of factors associated with overall survival (OS) in the univariate and multivariate analyses at multiple time points. ^†^(univariate), *(baseline multivariate), ^#^(preoperative multivariate), and ^§^(postoperative multivariate) indicate statistical significance, while a single symbol represents p<0.05, two symbols represent p<0.01, and three symbols represent p<0.001.

The results of the univariate and multivariate analyses of OS are summarized in **Figure 4**. Multivariate analysis revealed that two factors, namely, the PS and the TPS (**Figures 1D, E**), significantly affected OS(PS at all three time points and TPS at baseline and preoperative time points). In addition, the sum of the diameters of preoperative target lesions and pathological response (**Figure 1F**) were also found to be significant factors affecting OS at the preoperative and postoperative time point, respectively.

**Figure 5:**
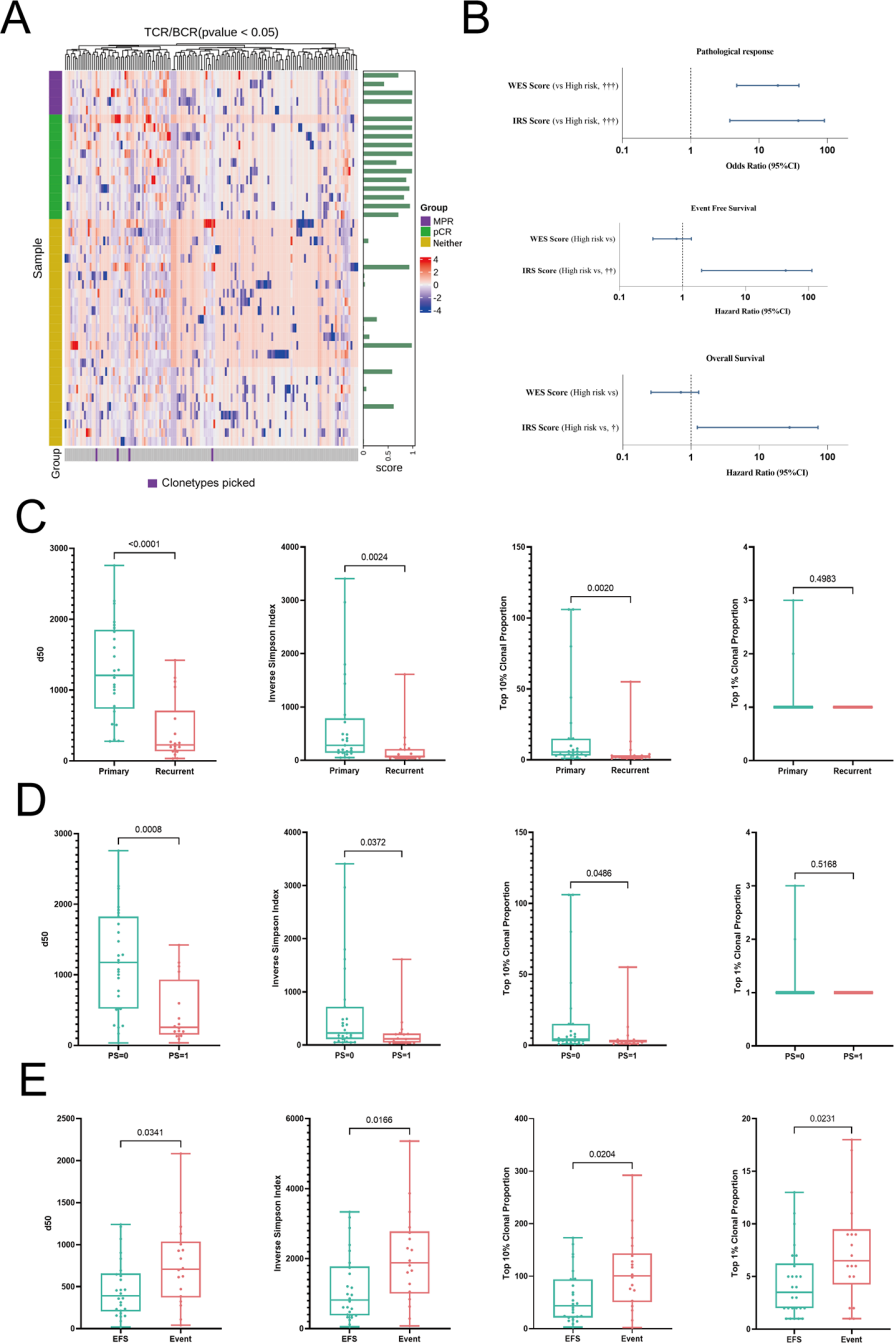
Immune repertoire and genomic landscape analysis. **(A)** Heatmap showing the distribution of immune repertoire sequencing (IRS) data divided into pathological complete response (pCR), major pathological response (MPR), or neither groups. The clonotypes picked in the IRS model are shown below the heatmap. **(B)** Univariate analysis of whole-exome sequencing (WES) and IRS logistic models for the prediction of major pathological response (MPR), event-free survival (EFS), and overall survival (OS). **(C)** Correlations of T-cell receptor (TCR) repertoire diversity indices (d50, inverse Simpson) and clonality indices (top 10% and top 1% clonal proportion) with primary tumor status (Mann–Whitney U tests). **(D)** Correlations of T-cell receptor (TCR) repertoire diversity indices (d50, inverse Simpson) and clonality indices (top 10%, top 1% clonal proportion) with performance status (PS) (Mann–Whitney U tests). **(E)** Correlations of B-cell receptor (BCR) repertoire diversity indices (d50, inverse Simpson) and clonality indices (top 10%, top 1% clonal proportion) with failure in event-free survival (Mann–Whitney U tests).

The distributions of the IRS and WES data are shown in **Figure 5A** and **Supplementary Figure 1A**, respectively. The genes selected in the WES and IRS models are listed in **Table 2**. Owing to insufficient sample overlap, only univariate analysis was performed, and the results are shown in **Figure 5B**. The WES model was found to be a significant factor for only pathological response (**Figures 1H**, **5B**). Among the top 20 genes with mutations, only *PABPC1* was correlated with the MPR, and no mutation significantly affected OS or EFS (**Supplementary Figures 1B, C**). In contrast, the IRS model demonstrated potential as a prognostic indicator for pathological response, EFS, and OS (**Figures 1I**, **5B**). In this model, four specific clonotypes were selected, including two BCR rearrangements (IGHV2-7000/IGHJ200, fold change (FC) = 2.653, and IGHV4-30-200/IGHJ400, FC = 0.251) and two TCR rearrangements (TRBV10-300/TRBJ2-200, FC = 1.548, and TRBV5-500/TRBJ1-500, FC = 3.059). A higher d50 index, inverse Simpson index, and top 10% clonal proportion of the TCR were correlated with primary tumor and a lower PS (**Figures 5C, D**). A higher d50 index, inverse Simpson index, top 1% clonal proportion, and top 10% clonal proportion of the BCR were correlated with failure to achieve event-free survival (**Figure 5E**).

**Table 2:** The genes picked in the WES and IRS models.

Model	Gene	p	Model	VGene	JGene	*p*
WES	AKAP9	0.018	IRS	IGHV2-70*00	IGHJ2*00	0.016
	CSMD3	0.045		IGHV4-30-2*00	IGHJ4*00	0.006
	ETV5	0.045		TRBV10-3*00	TRBJ2-2*00	0.004
	KIAA1549	0.018		TRBV5-5*00	TRBJ1-5*00	<0.001
	KMT2C	0.023				
	PABPC1	0.010				
	USP8	0.009				

The whole TNM staging system was not selected for multivariate analysis because of the results of the univariate analysis and clinical considerations. The results of the univariate analysis for TNM staging are provided in **Supplementary Table 2**.

## Discussion

In recent decades, the survival rate of patients with HNSCC has stagnated at approximately 50% (24), even at the expense of function and esthetics. In recent years, ICIs, especially neoadjuvant PD-1 blockade, have appeared to be the most promising approaches for improving survival and quality of life in patients with HNSCC (1, 20, 25). However, accurate factors supporting clinical decision-making before neoadjuvant therapy, surgery, and adjuvant therapy are needed. Therefore, in this study, we collected clinicopathological and sequencing data from patients across three studies to explore the factors associated with clinical decision-making for NACIT in patients with HNSCC at different time points. With a longer follow-up period and more events than those in similar prior studies (7, 26, 27) and integration with IRS data, we performed multivariate analyses, presented exploratory findings, and provided initial hints regarding the underlying mechanism.

MPR, a postoperative factor, and CPS, a baseline factor, are both regarded as independent predictors of EFS or OS in studies with relatively large sample sizes (12, 14, 28). In this study, we reported comparable response outcomes (MPR: 60.0%, pCR: 37.5%) and PD-L1 expression levels (CPS≥20: 26.3%) but lower survival outcomes (3-year EFS: 67.5%; OS: 74.6%) for primary patients than in other studies (MPR: 61.2–65.4%, pCR: 40.5–47.1%, and CPS>20: 21.1–48.1%; 3-year EFS: 78.8–89.0%; and OS: 90.9–91.9%) with similar sample sizes (104–152 patients) (12, 14, 28). These findings indicated that some independent predictors for survival have not been identified. In addition, the MPR was proven to be an independent predictor of EFS and OS in this study. The results also revealed that the TPS was an independent prognostic factor for both EFS and OS and that recurrent tumor was associated with significantly worse EFS rates than primary tumor was, which was expected.

PS, a baseline factor, was associated with all endpoints at almost all time points, implying that PS should be considered before any key decisions are made. With respect to immunotherapy alone, a PS greater than 1 was regarded as significantly associated with decreased OS and EFS rates in HNSCC patients after multivariate adjustment (29). In this study, we confirmed the significant correlation of PS with the response to immunochemotherapy for HNSCC. Furthermore, we reported that HNSCC patients with a PS of 1 had worse EFS and OS rates than did those with a PS of 0. Moreover, a higher PS was significantly associated with a worse pathological response after multivariate adjustment. These findings suggest that PS might not only reflect tolerance to therapy or tumors (30) but also be associated with antitumor immune activity.

Surgical strategies are decided mostly on the basis of radiological response, and recent studies have reported no significant differences in survival prognosis between patients who receive de-escalated surgery and those who receive radical surgery (12, 14, 28). The benefit of de-escalated surgery on quality of life has also been reported (4). Our findings support the use of de-escalated surgery for primary tumor but highlight critical concerns regarding the nodal surgical margin, which is an independent prognostic factor for EFS. Crucially, all positive margins (R+) in our cohort occurred in the neck dissection area of recurrent cases due to ENE involving the carotid artery. Since the primary site has attracted sufficient attention (12, 14, 28), our study supported that the regional nodal failure cannot be overlooked, and future therapeutic strategies should allocate greater focus to the management of the neck.

In addition to clinical and pathological factors, we performed univariate analyses on WES and IRS data. The WES model was insufficient to predict survival prognosis, which is consistent with the results of a previous study (27). While notably, in our analysis of the most frequent mutations, *PABPC1* was the only gene significantly correlated with MPR. *PABPC1* is a key regulator of mRNA stability and translation. Studies have shown that *PABPC1* is abnormally expressed in multiple cancer types, where it promotes tumor cell proliferation and epithelial-mesenchymal transition by stabilizing specific oncogenic mRNAs (31). Furthermore, recent research has demonstrated that *PABPC1* can alter the tumor immune microenvironment by regulating the stability or alternative splicing of immune-related transcripts (32). We hypothesize that mutations in *PABPC1* might disrupt tumor immune evasion or increase the sensitivity of HNSCC to NACIT, leading to a better pathological response.

For the IRS data, we constructed a model with the specific T/B-cell clonotype instead of the immune repertoire diversity and abundance indices, which are more commonly used (17, 19, 33). Although the IRS logistic regression model was initially constructed on the basis of pathological response, it was correlated with both EFS and OS. High initial frequencies of specific TCR clonotypes, such as TRBV5-5 (FC = 3.059) and TRBV10-3 (FC = 1.548), suggest that responders have a relatively large pool of tumor-reactive T cells before treatment. This finding are consistent with evidence that pretreatment TCR repertoire features predict clinical outcomes in immunotherapy (34). The baseline BCR clonotypes showed bidirectional predictive value. The higher level of IGHV2-70 (FC = 2.653) in responders may indicate an immune state that supports antitumor activity, possibly involving mature tertiary lymphoid structures (35). In contrast, the lower frequency of IGHV4-30-2 (FC = 0.251) in the MPR group suggests that high initial levels of certain B-cell clones are associated with poor response. While the biological role of this clonotype is not fully defined, its negative correlation with the MPR might reflect the presence of inhibitory or nonfunctional B-cell populations that limit NACIT efficacy (36). These results indicate that a successful response to NACIT depends on a favorable pretreatment immune profile, characterized by the presence of specific effector-related clones and fewer unfavorable clones.

In addition, most studies have focused on the relationship between T-cell receptors and the prognosis of patients receiving immunotherapy targeting PD-1, as CD8^+^ T cells are strongly correlated with the response. As mentioned above, both T-cell and B-cell clonotypes were included in the IRS logistic regression model, and high BCR diversity and clonality were correlated with failure in event-free survival. On the basis of studies on the mechanism of antigen cross-presentation by B cells with specific receptors to CD8+ T cells (17), our results further imply that humoral immunity might be as important as cellular immunity in tumor immunotherapy.

Also, we observed a correlation between favorable IRS features and a better PS as well as primary tumor. This finding relates our clinical and biological findings, namely, that a patient’s physical decline and long-term tumor burden may correlate with systemic immune exhaustion or senescence, characterized by a restricted TCR repertoire. Our results combined with those of a previous report (29) demonstrated that poor PS was associated with significantly worse prognosis. Recent trials have also shown that starting with immunotherapy can provide a substantial survival benefit compared with reserving it for second-line use (37). This concept is further supported by the known delayed separation of survival curves and late benefit patterns of immunotherapy (38), which implies that immunotherapy may work better in patients who can live long enough to develop lasting antitumor immune responses. Together, our results and these reports suggest that immunotherapy could be performed earlier when patients are healthier and have active immune systems rather than being reserved as a salvage option for patients with a compromised PS and long-lasting tumor burden, where systemic exhaustion of the immune system may limit its efficacy.

We acknowledge several limitations. First, as a pooled analysis combining retrospective and prospective cohorts, inherent heterogeneity in patient baselines may exist. Second, the sample size for the IRS analysis (N = 43) was relatively small due to the scarcity of available tissue, and the predictive model lacks an external validation cohort. Therefore, our findings regarding B-cell clonotypes should be interpreted as exploratory and hypothesis-generating. Future large-scale, prospective studies are needed to validate these translational insights.

## Conclusion

In this pooled analysis, we evaluated the prognostic value of clinicopathological factors and sequencing data for HNSCC patients who underwent NACIT. Clinically, we demonstrated that the PS was related to both response and survival. Additionally, de-escalation and radical surgery were associated with similar prognoses, while the positive surgical margin caused by extranodal extension significantly affected survival. Biologically, our IRS model based on specific clonotypes showed preliminary prognostic value and highlights the potential role of B-cell clonotypes and humoral immunity in NACIT responses. The observed correlation between the IRS indices and a better PS as well as primary tumor suggests that sensitization strategies for restoring systemic immunity are needed and demonstrate the advantages of performing NACIT earlier in patients.

## Data Availability

The datasets presented in this study can be found in online repositories. The raw TCR and BCR immune repertoire sequencing data from the recurrent HNSCC cohort have been deposited in the Genome Sequence Archive for Human (GSA-Human) at the China National Center for Bioinformation under accession number HRA017362 and are publicly available at https://ngdc.cncb.ac.cn/gsa-human/browse/HRA017362. The raw peripheral immune repertoire sequencing data from the primary HNSCC cohort have been deposited in GSA-Human under accession number HRA019325 and are publicly available at https://ngdc.cncb.ac.cn/gsa-human/browse/HRA019325. The whole-exome sequencing data analyzed in this study have been deposited in the Genome Variation Map (GVM) at the China National Center for Bioinformation under accession number GVM001469 and are publicly available at https://ngdc.cncb.ac.cn/gvm/getProjectDetail?project=GVM001469. De-identified clinical data and any additional information required to reanalyze the data reported in this article are available from the corresponding author upon reasonable request.
