# Supplementary material for: Integrating clinicopathologic factors with whole-exome sequencing and peripheral immune repertoire sequencing to optimize decision-making in neoadjuvant chemoimmunotherapy for HNSCC
Source: Front Immunol. 2026 Jul 30;17:1821734. doi: 10.3389/fimmu.2026.1821734 (PMC13470802; doi:10.3389/fimmu.2026.1821734)
Supplement: Supplementary file 1 [file DataSheet1.docx]

Supplementary Figure S1

A. The top 20 genes with mutation frequencies-Somatic(COSMIC).

B. Association between the top 20 mutated genes and pathological response.

C. Kaplan–Meier survival curves showing the EFS and OS of the top 20 mutated genes.
