# Supplementary material for: Integrating clinicopathologic factors with whole-exome sequencing and peripheral immune repertoire sequencing to optimize decision-making in neoadjuvant chemoimmunotherapy for HNSCC
Source: Front Immunol. 2026 Jul 30;17:1821734. doi: 10.3389/fimmu.2026.1821734 (PMC13470802; doi:10.3389/fimmu.2026.1821734)
Supplement: Supplementary file 2 [file DataSheet3.docx]

Supplementary Table S3. The univariate analysis for TNM staging

| Time point | Factors | Univariate | | | | | |
| --- | --- | --- | --- | --- | --- | --- | --- |
|  |  | Overall survival | | Event-free survival | | Pathological response | |
|  |  | HR (95%CI) | *p* | HR (95%CI) | *p* | OR (95%CI) | *p* |
| Baseline | **c(r)TNM** |  |  |  |  |  |  |
|  | T0 | ref | 0.863 | ref | 0.133 | ref | 0.073 |
|  | T1 | 0.377(0.037-3.468) |  | 0.106(0.180-1.968) |  | 0.214(0.014-3.370) |  |
|  | T2 | 0.000(0.000) |  | 0.000(0.000) |  | 0.143(0.008-2.517) |  |
|  | T3 | 0.392(0.094-1.643) |  | 0.227(0.073-0.704) |  | 0.048(0.005-0.488) |  |
|  | T4 or T4a | 0.588(0.174-1.989) |  | 0.403(0.165-0.982) |  | 0.183(0.021-1.585) |  |
|  | T4b | 0.502(0.084-3.008) |  | 0.219(0.044-1.090) |  | 0.057(0.004-0.817) |  |
|  | N0 | ref | 0.994 | ref | 0.649 | ref | 0.085 |
|  | N1 | 1.005(0.328-3.085) |  | 1.213(0.405-3.629) |  | 0.545(0.121-2.449) |  |
|  | N2 | 1.063(0.347-3.262) |  | 1.336(0.447-3.997) |  | 0.312(0.058-1.663) |  |
|  | N3 | 1.124(0.480-2.630) |  | 1.560(0.788-3.088) |  | 2.014(0.828-4.899) |  |
|  | III | ref | 0.882 | ref | 0.337 | ref | **0.002** |
|  | IVorIVA | 1.260(0.508-3.123) |  | 1.854(0.799-4.306) |  | 8.708(2.567-29.545) |  |
|  | IVB | 1.145(0.426-3.076) |  | 1.426(0.543-3.748) |  | 5.115(1.372-19.077) |  |
| Preoperative | **yc(r)TNM** |  |  |  |  |  |  |
|  | T0 | ref | 0.057 | ref | 0.300 | ref | **0.001** |
|  | T1 | 0.436(0.084-2.245) |  | 0.473(0.146-1.536) |  | 0.173(0.038-0.795) |  |
|  | T2 | 0.606(0.145-2.536) |  | 0.612(0.205-1.827) |  | 0.525(0.144-1.919) |  |
|  | T3 | 0.877(0.235-3.265) |  | 0.923(0.343-2.480) |  | 0.341(0.087-1.336) |  |
|  | T4 or T4a | 2.413(0.875-6.657) |  | 1.464(0.640-3.352) |  | 4.312(1.097-16.955) |  |
|  | T4b | 0.975(0.114-8.344) |  | 0.510(0.065-4.028) |  | 0.250(0.022-2.819) |  |
|  | N0 | ref | 0.512 | ref | **0.048** | ref | 0.064 |
|  | N1 | 1.796(0.646-4.988) |  | 2.931(1.280-6.709) |  | 5.079(1.249-20.654) |  |
|  | N2 | 0.979(0.281-3.409) |  | 1.937(0.751-4.997) |  | 1.306(0.385-4.431) |  |
|  | N3 | 1.708(0.716-4.074) |  | 2.281(1.046-4.972) |  | 2.667(0.954-7.457) |  |
|  | 0 | ref | 0.425 | ref | 0.257 | ref | **0.010** |
|  | I | 0.803(0.050-12.844) |  | 1.652(0.150-18.234) |  | 1.714(0.123-23.939) |  |
|  | II | 1.472(0.133-16.238) |  | 1.514(0.137-16.713) |  | 2.571(0.209-31.710) |  |
|  | III | 2.158(0.260-17.927) |  | 3.888(0.497-30.413) |  | 4.154(0.424-40.661) |  |
|  | IVorIVA | 3.764(0.489-28.974) |  | 5.187(0.684-39.338) |  | 18.857(1.926-184.631) |  |
|  | IVB | 3.053(0.382-24.418) |  | 4.520(0.583-35.068) |  | 8.400(0.870-81.080) |  |
